# Chromosome territories, X;Y translocation and Premature Ovarian Failure: is there a relationship?

**DOI:** 10.1186/1755-8166-2-19

**Published:** 2009-09-27

**Authors:** Sara Lissoni, Simona Baronchelli, Nicoletta Villa, Valeria Lucchini, Enrico Betri, Pietro Cavalli, Leda Dalprà

**Affiliations:** 1 Department of Neuroscience and Biomedical Technologies, University of Milan-Bicocca, via Cadore 48, 20052, Monza, Italy; 2 Medical Genetics Lab, Department of Clinical Pathology, HS. Gerardo, via Pergolesi 33, 20052, Monza, Italy; 3 Department of Clinical Pathology, HS. Gerardo, via Pergolesi 33, 20052, Monza, Italy; 4 Department of Pathology, Istituti Ospitalieri Hospital, Viale Concordia 1, 26100, Cremona, Italy; 5 Medical Genetics Lab, Istituti Ospitalieri Hospital, Viale Concordia 1, 26100, Cremona, Italy

## Abstract

**Background:**

Premature ovarian failure (POF) is a secondary hypergonadotrophic amenorrhea occurring before the age of 40 and affecting 1-3% of females. Chromosome anomalies account for 6-8% of POF cases, but only few cases are associated with translocations involving X and Y chromosomes.

This study shows the cytogenetic and molecular analysis of a POF patient came to our attention as she developed a left ovary choriocarcinoma at the age of 10 and at 14 years of age she presented secondary amenorrhea with elevated levels of gonadotropins.

**Results:**

Breakpoint position on X and Y chromosomes was investigated using Fluorescent In Situ Hybridisation (FISH) with a panel of specific BAC probes, microsatellite analysis and evaluation of copy number changes and loss of heterozigosity by Affymetrix^® ^GeneChip platform (Santa Clara, CA, USA). Patient's karyotype resulted 46, X, der(Y)t(X;Y)(q13.1;q11.223). X inactivation study was assessed by RBA banding and showed preferential inactivation of derivative chromosome. The reciprocal spatial disposition of sexual chromosome territories was investigated using whole chromosome painting and centromeres probes: patient's results didn't show a significant difference in comparison to normal controls.

**Conclusion:**

The peculiar clinical case come to our attention highlighted the complexity of POF aetiology and of the translocation event, even if our results seem to exclude any effect on nuclear organisation. POF phenotype could be partially explained by skewed X chromosome inactivation that influences gene expression.

## Background

Translocations involving X and Y chromosomes occur rarely in the human population and are often associated with anomalies of gonadal development.

A possible mechanism resulting in chromosomal rearrangement could be an error in recombination between X and Y, normally restricted to the pseudoautosomal regions (PAR1 and PAR2), but occasionally it can occur outside these regions due to homologous sequences between X and Y chromosomes [1]. This recombination error can cause the transfer of Yp sequences, including SRY, on the terminal X short arm. This phenomenon is associated with variable sexual phenotype: fertile males and females, 46, XX maleness and in rare case 46, XX true hermaphrodites [2].

Xq and Yq interchanges are less frequent and only few cases of females with Xq;Yq translocation have been reported in literature [3-6].

Derivative chromosome maintaining X inactivation center (Xq13.2) is affected by skewed X chromosome inactivation but the spreading of inactivation to the translocated region could be incomplete and discontinuous, inducing gene expression imbalance [7]. In translocations between Yp and Xq the spreading of X inactivation on the translocated Yp segment carrying the SRY gene could account for the incomplete masculinisation which is occasionally observed in individuals with this kind of translocation [8].

Moreover in literature it has been proposed that chromosomal rearrangements could induce a position-effect on genes flanking the breakpoint, causing variation of normal gene expression by disrupting long-range regulatory transcriptional elements [9,10]: maybe this phenomenon is due to the removal of regulatory elements or to the alteration of local chromatin structure induced by the rearrangement, modifying the expression of adjacent genes as observed for other diseases [11].

In the last years many works in literature attributed a great importance to the spatial organisation of chromatin within the nucleus in the process of gene expression [12]. In human cells each chromosome is confined to a discrete region: the chromosome territory physically separated during interphase [13,14]. Local chromatin architecture is linked to gene expression: inactive regions are more condensed while active ones decondensed [15]. The neighbourhood position of chromosomes in the nucleus could be of great importance for some cellular processes as gene expression or rearrangement induction [16].

In the present study we propose an investigation of the possible mechanism that underlie the pathological phenotype in a POF patient who developed an ovarian choriocarcinoma at the age of 10. The karyotype revealed a derivative Y chromosome. First we characterised the translocation through a cytogenetic-molecular study to identify the breakpoints and the possible role of the derivative Y chromosome in POF aetiology.

## Results

### Case report

Family history was negative for birth defects, mental retardation, congenital malformations, or consanguinity. Pregnancy history was normal and at birth the patient had no dysmorphic features. At the age of 10 she complained of abdominal pain: pelvic and abdominal ultrasound revealed a left ovarian tumor mass that was identified as a pure non gestational choriocarcinoma (10 × 8 × 6 cm, 240 g). The mass was partially encapsulated and adherent to the Fallopian tube (6 × 0.5 cm), notably hemorrhagic. At laparotomy, the left oophorectomy was done, then the patient started the chemotherapy (five cycles of Cisplatin, Etoposide, and Bleomycin, PEB).

**Figure 1:**
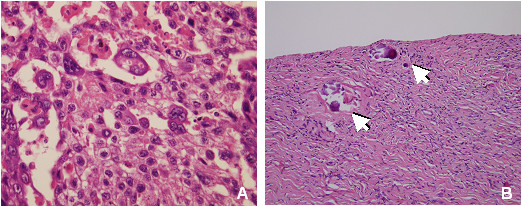
**Histological analysis**. Section of choriocarcinoma, left ovary (A): biphasic pattern of sheets of mononuclear cytotrophoblasts and multinuclear syncytiotrophoblasts located near hemorrhagic and necrotic areas. There are no elements suggesting other germ cell tumors (HE staining). Right ovary section (B): fibrous parenchyma, absence of follicles and neoplastic alterations. Arrows: microcalcifications (HE staining).

Histological examination revealed a choriocarcinoma with solid neoplastic areas, extensive necrosis and hemorrhage, composed by a core of cytotrophoblast cells surrounded by irregular rims of syncytiotrophoblast cells. In some cells nuclei were central, small and hyperchromatic, while in others nuclei were large and vescicular and there was evidence of mitotic activity (Figure 1A).

The patient had menarche at 13 years of age, subsequent menstrual cycles were irregular than stopped after few months, showing high levels of gonadotropins (FSH = 104 mIU/ml; LH = 34 mIU/ml).

Physical analysis showed no dismorphic features, regular mammary development, normal height and weight values (1,58 mt, 58 kg). She hadn't any ambiguity in inner and outer genitals, and she had no ovarian stigmata associated with Turner Syndrome.

She was healthy until 2003 when she had an unexplained weight gain. Laboratory examination showed TSH (thyroid-stimulating hormone) levels of 17 mIU/ml and anti-thyroid peroxidase antibodies levels of 611 IU/ml, serum IgA levels were 3 mg/dl. Thyroid scintigraphy analysis and ultrasound examination allowed to diagnose a Hashimoto's thyroiditis.

At the age of 16, the patient underwent preventive right ovary laparoscopic oophorectomy because of the increased risk of ovary tumor due to the presence of the Y chromosome. Histological examination of the right gonad showed signs of fibrous tissue, stromal cells and microcalcifications; there were no germ cells neither evidence of neoplastic tissue (Figure 1B). These dismorphic characteristics probably are connected to partial X monosomy. Actually the patient is in good health.

### Cytogenetic and molecular analysis

**Figure 2:**
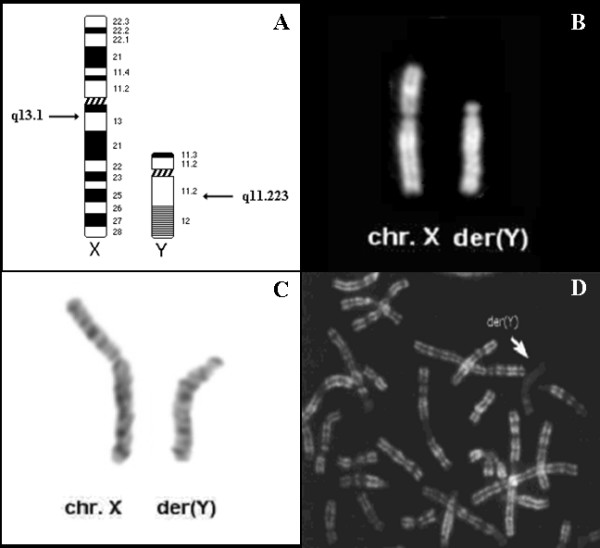
**Cytogenetics analysis**. Ideogram of X and Y chromosomes: arrows indicate the breakpoint position in Xq13.1 and Yq11.223. (A). Conventional cytogenetic analysis: pair of sex chromosomes Q-banded (B) and G-banded (C). R-banded metaphase spread from patient's lymphocytes after BrdU incorporation in late S-phase. The derivative Y chromosome (arrowed) shows late replication (D).

**Figure 3:**
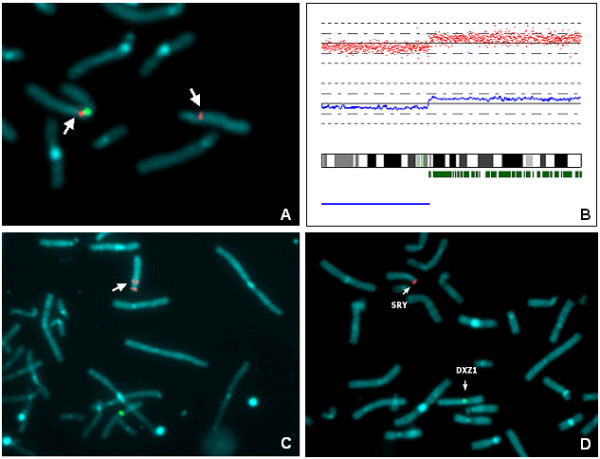
**Molecular-cytogentics analysis of X and Y chromosome breakpoint**. Partial metaphase plate after FISH with RP11-368D24 probe (Xq11.1). The signal (red) was detected both on der(Y) and X chromosome (arrows). CEP X probe (Cytocell, green) was used to identify X chromosome (A). Location of disomic SNP calls (small green vertical bars) is shown along the long arm of X chromosome ideogram. The blue bar below the ideogram indicates the region of monosomy. In the upper histogram the log_2 _intensity ratio for each SNP locus (red dots) is represented and the blue line below shows the averaged log_2 _values. Breakpoint is localised between SNP_A-1753661 (nt.68.182.937) and SNP_A-1659250 (nt.69.788.317) in Xq13.1(B). There is a discrepancy between the results obtained through FISH using BAC probes and Affymetrix^® ^analysis of SNPs on X chromosome (A, B). Partial metaphase plate after FISH with RP11-65G9 (Yq11.223) probe. The signal (red) was detected on der(Y) (arrow). LSI SRY SpectrumOrange/CEPX Spectrum Green probe (Vysis, Abbott Molecular, Illinois, USA) was used as control probe (C). Dual-color FISH analysis on chromosome spreads (partial metaphase) with LSI SRY SpectrumOrange/CEPX SpectrumGreen probes. Arrows: normal and derivative chromosome showed specific hybridization signals that are localised correctly (D).

QFQ and GTG-banding of lymphocytes identified a derivative Y chromosome due to a de novo Xq;Yq unbalanced translocation (Figure 2A-C): this rearrangement was studied by FISH using a panel of BAC probes and microsatellite polymorphic markers (Additional file 1). Therefore X chromosome breakpoint was initially mapped on Xq11.1 (Figure 3A). To complete the previous mapping data and to identify cryptic copy number changes (gain or loss) on other autosomes, we performed an Affymetrix^® ^GeneChip analysis of SNPs (Figure 3B). LOH and SNP array analysis identified the breakpoint on chromosome X in Xq13.1. The array didn't show any significant copy number changes on other chromosomes, so no other genomic alterations could be responsible for the pathologic phenotype. There was a clear discrepancy between the localisation of the breakpoint on X chromosome obtained by FISH and by Affymetrix^® ^analysis. BAC probes localisation has been evaluated on normal X chromosome and it has been found correct. The discrepancy between the breakpoint localisation on X chromosome obtained by FISH and by Affymetrix^® ^analysis can be explained by a further intrachromosomal rearrangement occurred during translocation event.

The low-copy repeat/nonallelic homologous recombination is the mechanism that underlie the formation of chromosome complex rearrangements. An intrachromatid loop of inverted repeats could result in inversion and so this mechanism could explain our results (Additional file 2) [17]. Furthermore X chromosome contains a disproportionately high representation of inverted repeats that could be at the base of this process [18].

Moreover, cytogenetic and molecular analysis identified the breakpoint site in Yq11.223 (Figure 3C).

Fluorescent in situ hybridization (FISH) performed with Sex-determining Region Y (SRY) gene probe revealed the presence of the gene on the derivative chromosome Y (Figure 3D), but sequencing analysis didn't reveal any mutation on the coding region.

Replication banding analysis on metaphases from lymphocytes showed non random inactivation. In 90% of metaphases the derivative Y chromosome was late replicating (Figure 2D). Moreover the derivative Y chromosome appeared as a more intensively stained heterochromatic body with a more condensed, defined and regular shape compared to normal X chromosome which instead was more spread and characterised by a less defined surface (Additional file 3) [19]. Additionally, derivative chromosome was preferentially found in association to the nuclear membrane in 66% of analysed interphase nuclei, in intermediate position in 32% and in central position in 2% of nuclei. Percentages were compared to the inactive X chromosome position in a normal female control (external 78%; intermediate 21%; central 1%): there was no significant difference between the patient and the control (χ^2 ^test; p = 0.164).

### Bioinformatic analysis

RepeatMasker  analysis of breakpoint sequences on X chromosome revealed a high percentage of repeat elements (SINEs 16,96%; LINEs 31,84%; LTR elements 4,33%). The same analysis on Y chromosome revealed that the breakpoint fell in a cluster of repetitive elements (SINEs 2.77%; LINEs 46.15%; LTR elements 33.39%). These percentages differ from whole genome composition (SINE 13.14%; LINE 20.42%; LTR 8.29%), but for X chromosome the difference is not significant (p = 0.188), while Y chromosome showed a percentage bias (p = 0.000) that is probably due to its own general genomic composition. BLAST search (Basic Local Alignment Search Tool - ) was performed to identify eventual sites of sequence similarity on breakpoint sites of X and Y chromosomes. This research allowed to exclude gene fusion formation and the presence of sequence homology regions between X and Y chromosome.

### Chromosome territory analysis

Spatial distribution of sexual chromosome territories was investigated in 100 interphase nuclei for both the patient (lymphoblasts) and each of the 8 adult controls considered (lymphocytes). Controls are phenotipically normal and they are composed by 4 males and 4 females with regular menstrual cycles. Data were divided in three groups based on the measure of the distance between chromosome territories as summarised in Table 1.

**Table 1:** Reciprocal distribution of X and Y chromosome territories in thr patient, male and female controls.

	**Chromosome territory distance**			
	**≤ 30%**	**30-50%**	**>50%**	**Total**
**Patient**	26 26%	29 29%	45 45%	100

**Male controls** **(n = 4)**	101 25.25%	148 37%	151 37.75%	400

**Female controls** **(n = 4)**	99 24.75%	155 38.75%	146 36.5%	400

**Pooled controls** **(n = 8)**	200 25%	303 37.9%	297 37.1%	800

X and Y chromosome reciprocal distribution in male controls didn't reveal any significant difference compared to the disposition of X chromosome territories in female controls (p = 0.876), so they were pooled into a unique group of control.

**Figure 4:**
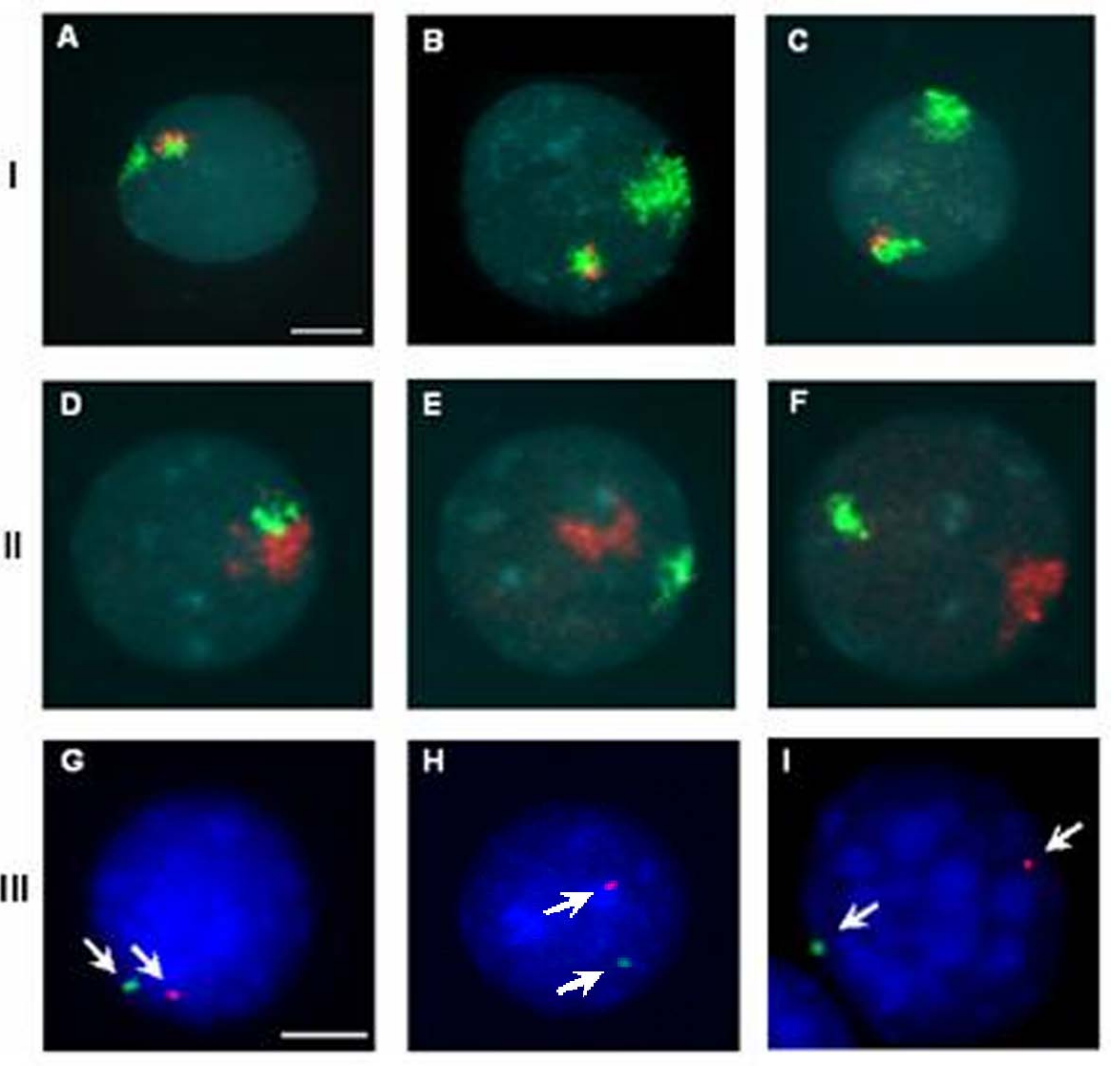
**Chromosome territory analysis**. FISH on interphase nuclei to detect reciprocal disposition of chromosome territories (line I and II). FISH was performed using WCPX (green in I/red in II) and WCPY (red in I/green in II). The images represent the three groups of chromosome territory disposition in the patient (I) and in male controls (II): distance smaller than 30% (A, D), distance between 30% and 50% (B, E) and distance more than 50% (C, F) of nuclear diameter. FISH on interphase nuclei to visualise centromere disposition of patient's sexual chromosome (line III) using CEPX (green) and CEPY (red) probes. The images represent the three disposition groups of centromeres (arrows) within the nucleus: signals positioned at a distance smaller than 30% (G), between 30% and 50% (H) and more than 50% (I) of nuclear diameter. Bar = 10 μm.

The comparison between the distribution of chromosome territories of the patient and the group of controls (Figure 4A-F, Table 1) showed no difference (p = 0.184): these preliminary observations suggested that the relative position of X chromosome and derivative Y chromosome wasn't different from normal controls. Statistical analysis is summarised in Additional file 4.

Territory architecture was evaluated visualising the localisation of centromere sequences within the nucleus. Normal controls were composed by a suspension of uncultured amniocytes recruited from routine diagnostic activity for rapid research of aneuploidies (13, 18, 21, X and Y chromosomes) associated with foetal karyotype. Amniocytes were processed with the same protocol used for lymphoblasts. We chose amniocytes because they have an early differentiation stage that can be related to the lymphoblasts one, furthermore FISH analysis on amniocytes was easy to achieve.

X and Y centromere distribution in interphase nuclei was evaluated on normal males nuclei (n = 5, 250 nuclei) and on 50 interphase nuclei (lymphoblasts) for the patient (Figure 4G-I). χ^2^-test revealed no significant difference in the spatial disposition of centromeres between our patient and the control group (p = 0.318). Data are summarised in Table 2, statistical analysis in Additional file 4. These results suggest that this specific X;Y translocation doesn't modify nuclear architecture and therefore POF phenotype could not be related to a change in chromosome territory organisation.

**Table 2:** Reciprocal distribution of X and Y centromeres in the patient, male and female controls.

	CEP* X; CEP Y distance			
	**≤ 30%**	**30-50%**	**>50%**	**Total**
**Patient**	9 18%	17 34%	24 48%	50

**Male Controls** **(n = 5)**	65 26%	91 36.4%	94 37.6%	250

*CEP = Chromosome Enumeration Probe.

## Discussion

Here we report the cytogenetic and molecular analysis of a *de novo *X;Y unbalanced translocation involving chromosome bands Xq13.1 and Yq11.223.

X microsatellite analysis of parents allowed to identify the paternal origin of derivative Y chromosome (data not shown): it originated from an error occurred during male meiotic cellular division, that led to an Xq and Yq interchange, a rare event, as reported in literature [3-6].

In the patient, the mechanism through which Y euchromatin has been transferred to Xq, is different from traditional mechanisms -NAHR, Non-Allelic Homologous Recombination- [20]: bioinformatic analysis didn't show any similarity between X and Y breakpoint in Xq13.1 and in Yq11.223, therefore our data could not explain the mechanism promoting the rearrangement. Anyway the low-copy repeat/nonallelic homologous recombination mechanism can result in genomic inversion [17], as supposed previously: the parental X chromosome could have been subjected to inversion process and then to the translocation event, which unfortunately can not be proven.

First of all, the patient had a monosomy of X chromosome until the band Xq13.1, but nevertheless she hadn't any typical characteristic of Turner Syndrome.

Chromosomal breakpoints at different positions on one of X chromosomes usually induce gonadal failure, due to the interruption of genes involved in ovarian development, as many of these genes seem to escape X inactivation, so an eventual haploinsufficiency could bring to the pathological phenotype.

Several works described two distinct regions on Xq involved in ovarian activity: one at Xq13-21 (POF2) and the other at Xq23-q28 (POF1). Bioinformatic analysis of the breakpoint region on Xq allowed to characterise the genomic region, in particular the breakpoint fell in the first critical cluster (POF2), a gene rich region. KIF4A (Kinesin Family Member 4A), mapping in this site, is an interesting gene because it is involved in cellular division. This gene codifies for a protein that acts in molecular movements along microtubules influencing many cellular processes as spindle regulation and chromosomes segregation during mitosis [21]. Therefore an eventual functional alteration of KIF4A could be associated with an altered division and the depletion of germ cells during embryogenesis; ovarian failure might be caused by the precocious loss of oogones and subsequent gonadal deficiency. Anyway, the supposed role of KIF4A on the pathological phenotype of our patient remains to be clarified.

Scientific literature suggests that genes interruption is not the major cause of pathological phenotype [9], in particular the breakpoint could cause POF through modification of long-range transcriptional regulative control, modifying the expression of genes flanking the breakpoint [10].

The complex features of the patient induced us to explore other possible cellular mechanisms involved in the pathological phenotype, trying to understand more globally the mechanisms underlying POF phenomenon. Recently, many works in literature show that spatial nuclei organisation and positioning of chromosomes might be very important in the regulation of gene expression and cellular processes: changes in genome organisation in diseased cells compared to normal ones could be associated to some pathologies [22]. The reciprocal position in the nucleus of chromosome X and derivative Y territories was investigated to identify any eventual correlation between nuclear spatial perturbation and pathologic effects on the patient.

The evaluation of the reciprocal signal disposition in interphase nucleus is an efficient method to evaluate the disposition of CT because in this way it is possible to estimate the modification of nuclear organisation even without analysing the radial distribution. If there is a perturbation in the CT disposition (that could influence gene expression) it can also be found with the reciprocal evaluation of painting distances (only the reference point changes). The repositioning of chromosomes in cells associated with a pathology was found for the first time in cortical neurons of brain samples derived from epilepsy patients compared to normal ones [23].

In literature several works observed that the comparison between data collected from 2D preparations and 3D-preserved nuclei confirm the validity of our type of analysis: the organisation of the nucleus is not significantly altered [24-26]. In conclusion, our method allows a large scale and fast chromatin organisation study and our results are fully reliable and valid according to literature.

The analysis didn't reveal any significant modifications of nuclear space organisation of investigated territories in the patient compared to normal controls (p = 0.184).

The importance of genome organisation in the nucleus is not only significant related to the chromosome territory disposition, but also in their internal structure [27].

Thus, the genomic composition of derivative chromosome could induce an internal organisation change of chromosome territory, that we tried to detected by measuring centromere reciprocal disposition in the interphase nucleus.

Centromeres are highly repetitive α-satellite sequences and are prone to form chromatin repression structures that can influence gene expression regulation of other regions by a long-range mechanism, resulting in heterochromatic zones. Thus the nature of translocated regions is very important in the whole phenomenon of translocation, because they can influence the inner structure of chromosome territory, modifying its functionality [13].

However, centromere distribution results didn't show significant change in the nuclear disposition so it's possible to reject the above-mentioned hypothesis.

The deleterious effect on expression of genes located on the derivative chromosome could be related to skewed X inactivation mechanism. Different patterns of inactivation of the derivative X carrying Yp sequences explain different sexual phenotypes such as male, hermaphrodite or female because of the spreading of X inactivation into Y chromatin or by positional effect [8]. Even if the derivative X chromosome is always normally inactivated, many genes (15%) escape X inactivation [28] and their presence in a single copy in a female with an X chromosome abnormality is harmful. X inactivation analysis of the patient showed a non random X inactivation pattern identified by late replication of derivative Y chromosome in the 90% of analysed metaphases. Therefore positional analysis of derivative chromosome in interphase nuclei showed that it was preferentially located close to the nuclear membrane in 66% of nuclei (Additional file 3), a position which is typical of inactive X chromosome, according to literature [29]. Moreover the morphological analysis on nuclei images by visual evaluation revealed that the derivative Y chromosome had a more condensed and defined shape compared to normal X chromosome which is more spread showing an irregular surface.

A high percentage of 46, XY female develops gonadal tumors as gonadoblastoma or dysgerminoma. In general only 20% of XY females show mutation or alteration of SRY gene [30]. Sequence analysis of SRY gene in our patient showed no mutation. We hypothesised that the X;Y translocation might reduce SRY functionality inducing a variation in the expression level, especially through the preferential inactivation of the derivative Y chromosome. During embryonic development X inactivation occurs at the stage of pre-implantation embryo [31], while SRY expression is detected only at 41 days after conception [32], so it's possible that SRY couldn't express its function in the embryo, inhibiting the masculinisation.

Taken together these results suggest that a skewed X inactivation could bring to a change in normal gene silencing, in relation to a possible change in chromosome territory organisation.

In females the presence of Y-chromosome-derived material increases the risk of malignant germ cell tumors [33]. Our patient developed a choriocarcinoma, a rare primary ovarian tumor, with an estimated incidence of 1 in 369.000.000, with approximately 40 cases described in literature [34]; gonadoblastoma and dysgerminoma in association with Y chromosome material are more frequent [35]. Non-gestational choriocarcinoma is not associated with gestational events and germ cell tumor differentiates towards trophoblastic structures and is composed by mixed germ cell populations with other histological origins, as observed in the patient. Further investigations on the tumor weren't possible because we couldn't retrieve the surgical specimens. Anyway these results accorded with the presence of germ cells in the patient's ovary because this type of tumor origins from germinal material, even if the histological analysis of the right fibrous ovary showed no germ cells, maybe due to ovarian failure.

It's still unclear the reason why the patient developed this rare type of tumor and in which way the translocation involving sexual chromosomes could be responsible for this malignancy.

## Materials and methods

### Histology

The surgical specimens were fixed in formalin and examined with standard methods after paraffin embedding sectioning and Hematoxylin Eosin staining.

### Cytogenetic analysis

Metaphase-chromosome spreads were obtained from phytohaemagglutin-stimulated peripheral blood lymphocytes. Chromosomes were QFQ-banded using quinacrine mustard and slides were mounted in McIlvaine Buffer. A minimum of 20 cells were analysed for karyotype and further 50 cells assessed to exclude sex chromosomes mosaicism. The karyotype was reconstructed following the guidelines of the International System for Chromosome Nomenclature 2009 (ISCN 2009). X inactivation was assessed by means of late-replication study, following standard method of BrdU incorporation in lymphocyte cultures.

### Fluorescent in situ Hybridization

Metaphase spreads were obtained from EBV-transformed lymphoblastoid cell lines, provided by Genetics Lab of Galliera Hospital (Genoa, Italy). 200 μl of Colcemid (10 μg/ml, Roche) were added to culture medium and after 15 minutes cells were subjected to hypotonic treatment (1:1 sodium citrate 1%:KCl 75 mM) for 13 minutes at 37°C and then fixed with 3:1 methanol:acetic acid solution at room temperature. Chromosome spreads were dropped onto glass slides and allowed to dry.

Fluorescent In Situ Hybridization (FISH) analysis was carried to define the breakpoint using several BAC probes (supplied by Wellcome Trust Sanger Institute, Hinxton, Cambridge, UK) localised in Xq and Yq. BAC probes used in this study are summarised in Additional file 1: selection and localisation of probes were established by the consultation of UCSC Genome Browser  and NCBI .

Probes were labelled by nick-translation with digoxigenin (DIG)-11-dUTP (Roche, Basel, Switzerland) as suggested by the manufacturer's instructions, precipitated and redissolved in hybridization solution (50% formamide, 10% dextran sulphate, 2 × SSC).

Chromosome slides were incubated for 90 min at 90°C on a hot plate, then treated with a 0.005% pepsin/0.01 M HCl solution for 30 min at 37°C. Slides were washed in 1 × PBS (Phosphate Buffered Saline), and immersed first in a Buffer solution (1 × PBS, 0.05 M MgCl_2_) for 5 min at room temperature and then in 4%Paraformaldeide (1 × PBS, 0.05 M MgCl_2_, 4%Paraformaldeide) for 5 min at room temperature. Slides were dehydratated with an ethanol series (70%, 85%, 100%) and air dried at room temperature.

Probe was applied on slide and co-denaturated on HYBrite (Abbott Molecular, Illinois, USA) at 72°C for 5 minutes. Hybridization was performed at 37°C over night in a moist chamber. Post-hybridization washes consisted in: three washes in 0.1 × SSC pH7 at 60°C (5 min each) and the subsequent incubation in a blocking solution (3%BSA, 4 × SSC, 0.1%Tween20) in moist chamber for 30 min at 37°C. Detection of digoxigenin probes was obtained by means of 1 μg/ml anti-DIG Rodaminate antibodies (Roche, Basel, Switzerland) after incubation in a moist chamber for 30 min at 37°C and three washes in 0.1%Tween20/4× SSC pH7 at 42°C (5 min each). Chromosomes were stained with DAPI (4',6-diamidino-2-phenylindole). For each FISH experiment, a mean number of 10 metaphases were analysed for the presence/absence of probe signal in normal and derivative chromosomes.

FISH analysis was also used to determine the presence of SRY gene using SRY/CEP X probe (Vysis, Abbott Molecular, Illinois, USA). Chromosome territories were studied by FISH on interphase nuclei with WCPX digoxigenin-labelled (Al-Technologies, Arlington, VA, USA); WCPY (Vysis, Abbott Molecular, Illinois, USA), CEPX and CEPY (Vysis, Abbott Molecular, Illinois, USA), according to the manufacturer's instructions.

All digital images were captured using a Leica DM 5000B microscope (Leitz, Germany) equipped with a CCD camera and analysed by means of Chromowin software (Casti Imaging, Venezia, Italy).

### Chromosome territory analysis

Chromosome territory position was investigated by FISH analysis performed using: WCPX (Whole Chromosome Paint of X; Altechnologies) probe modified by Digoxigenin (DIG)-11-dUTP and then detected by Anti-DIG-FITC (Roche); WCPY (Whole Chromosome Paint of Y; Vysis, Inc.) probe directly labelled and detected in Spectrum Orange. For centromeres study, FISH was performed with: CEPX probe specific for α-satellite sequences of chromosome X (Spectrum Orange); CEPY probe specific for α-satellite sequences of chromosome Y (Spectrum Green). These two probes belong to the Aneuvysion^® ^diagnostic kit (Vysis, Inc.). FISH experiments were performed according to the manufacturer's instructions.

### Image analysis

Image analysis was performed on interphase nuclei in two-dimension. Cell nucleus images were analysed using ImageJ [36], free access by NIH . Territories and centromeres of interest were manually defined, then the program calculated the mass centroid coordinates of each area [37]. The program allowed us to determine the distance between identified centroids and to normalise it by the radius of a circle of equal area to the nucleus (Additional file 5). The reciprocal signal disposition was classified as distance minor of 30%, between 30 and 50% and major of 50% of nucleus diameter.

X chromosome inactivation analysis by chromosome territory disposition in the nucleus was evaluated dividing each nucleus in three concentric circles of equal area and then the position of derivative and X chromosomes was analysed (Additional file 3). The inactivation of X and derivative Y chromosome was investigated by visual morphological evaluation of collected images (n = 100).

Statistical analysis of data by χ^2^-test was carried out using *primer.exe *program, version 1.0.

### Microsatellite analysis

Genomic DNA was extracted from whole blood using standard protocols. Y Chromosome AZF Analysis System (Promega, Madison, WI, USA) was used for an initial definition of the breakpoint region. Further analysis were performed with other microsatellites on X and Y chromosomes to better localise the breakpoint site (Additional file 1). All the microsatellite markers used in this study showed more than 70% heterozygosity and they were selected consulting UCSC Genome Browser .

### SRY sequencing

Genomic DNA was extracted from lymphoblast cultures by phenol-chloroform standard protocol. SRY amplification and mutational analysis were performed by PCR reaction using primers described in Additional file 6. Each PCR reaction was carried in a total volume of 25 μl, including 5× PCR buffer, 10 mM MgCl_2_, 10 mM dNTPs, forward and reverse primers (15 pm/μl each), 5 U/μl Taq-Polimerase and 100 ng DNA. Sequencing reactions were performed by Consorzio di Genetica Molecolare Umana (Monza, Italy) with ABI PRISM 3130 Genetic Analyzer (Applied Biosystem, Carlsbad, CA, USA).

### Affymetrix^® ^Mapping Array

The SNP mapping assay was performed by Genopolis (University of Milan-Bicocca, Milan, Italy) using a Affymetrix^® ^Mapping 50k_Xba248 platform, according to the protocol supplied by the manufacturer (Affymetrix^®^, Inc., Santa Clara, CA, USA). The array was scanned and the signal intensity was measured using GCOS (Gene Chip Operating System). Data were analysed using CNAG (Copy Number Analyzer for GeneChip) software version 1.0, evaluating DNA copy number and LOH.

## Competing interests

The authors declare that they have no competing interests.

## Authors' contributions

LD has made substantial contribution to the conception and design of this work, analysis and interpretation of data and she has revised critically the manuscript. SL and SB have carried out the molecular, molecular-cytogenetic studies and data analysis and they have drafted the manuscript. NV has been involved in the cytogenetic characterisation and she participated in the design and coordination of this work. VL and EB have carried out the histological analysis. PC was responsible for patient examination and genetic counselling. All the authors have read and approved the final manuscript.

## Supplementary Material

Additional file 1 **List of BAC probes and microsatellites used in the study**. Localisation and results summary of BAC probes and microsatellite used in molecular and molecular-cytogenetics analysis. Click here for file

Additional file 2 **Visual explication of possible rearrangement occurred during translocation process**. The low-copy repeat/nonallelic homologous recombination can result in complex rearrangements explaining our results discrepancy. Click here for file

Additional file 3 **X inactivation analysis by chromosome territory disposition**. A. Visual explication of the method used to evaluate derivative chromosome position in the nucleus. B. Image of X and der(Y) chromosome territories in interphase nuclei. Click here for file

Additional file 4 **Statistical analysis of chromosome territory (CT) and centromere distribution in interphase nuclei**. A. The table summarise the statistical analysis (χ^2 ^test) performed on the results of CT distances (X, Y and derivative Y chromosomes) in interphase nuclei. B. The table summarise the statistical analysis (χ^2 ^test) performed on the results of X and Y centromere reciprocal distribution in interphase nuclei. Click here for file

Additional file 5 **Chromosome territory image analysis**. Graphic representation of chromosome territory image analysis. Click here for file

Additional file 6 **Sequences of forward and reverse primers used in PCR reaction for SRY gene sequencing**. Primer details for SRY sequencing. Click here for file
