# Supplementary material for: Chromosome territories, X;Y translocation and Premature Ovarian Failure: is there a relationship?
Source: Mol Cytogenet. 2009 Sep 27;2:19. doi: 10.1186/1755-8166-2-19 (PMC2761935; doi:10.1186/1755-8166-2-19)
Supplement: Additional file 1 — List of BAC probes and microsatellites used in the study. Localisation and results summary of BAC probes and microsatellite used in molecular and molecular-cytogenetics analysis. [file 1755-8166-2-19-S1.DOC]

**Additional file 1**

|  | **Band** | **Localisation** | **der(Y) signal** |
| --- | --- | --- | --- |
| **RP11-65G9** | Yq11.223 | 21.557.573-21.722.451 | + |
| **G66594** | Yq11.223 | 21.737.897- 21.737.979 | + |
| **G66595** | Yq11.223 | 21.779.911- 21.780.143 | - |
| **RP11-120E18** | Yq11.223 | 21.816.712- 21.990.381 | - |
| **DXS6785** | Xq11.1 | 64.679.211-64.679.601 | - |
| **RP11-368D24** | Xq11.1 | 64.705.365- 64.905.366 | + |
| **RP11-434B14** | Xq11.1 | 64.811.739- 64.989.684 | + |
| **RP11-30M20** | Xq12 | 65.188.796- 65.272.196 | + |

**List of BAC probes and microsatellites used in the study.** Localisation of BAC probes and microsatellites used to identify chromosomes breakpoints. In the last column results on der(Y) chromosome are reported (+ presence of signal; - absence of signal).
