# Supplementary material for: Chromosome territories, X;Y translocation and Premature Ovarian Failure: is there a relationship?
Source: Mol Cytogenet. 2009 Sep 27;2:19. doi: 10.1186/1755-8166-2-19 (PMC2761935; doi:10.1186/1755-8166-2-19)
Supplement: Additional file 2 — Visual explication of possible rearrangement occurred during translocation process. The low-copy repeat/nonallelic homologous recombination can result in complex rearrangements explaining our results discrepancy. [file 1755-8166-2-19-S2.DOC]

**Additional file 2**


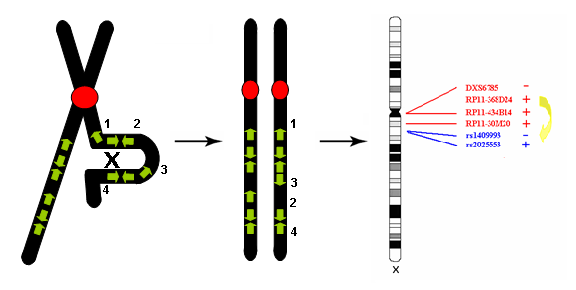


**Visual explication of possible rearrangement occurred during translocation process.** This figure shows the possible mechanism that underlie the discrepancy between FISH and Affymetrix® analysis: the low-copy repeat/nonallelic homologous recombination-based mechanism for genomic rearrangements. It is possible that an intrachromatid loop due to inverted repeats can result in inversion (left). The supposed inversion on X chromosome (right) could lead to different results between the two types of analysis.
