# Supplementary material for: Chromosome territories, X;Y translocation and Premature Ovarian Failure: is there a relationship?
Source: Mol Cytogenet. 2009 Sep 27;2:19. doi: 10.1186/1755-8166-2-19 (PMC2761935; doi:10.1186/1755-8166-2-19)
Supplement: Additional file 3 — X inactivation analysis by chromosome territory disposition. A. Visual explication of the method used to evaluate derivative chromosome position in the nucleus. B. Image of X and der(Y) chromosome territories in interphase nuclei. [file 1755-8166-2-19-S3.DOC]

**Additional file 3**

**X inactivation analysis by chromosome territory disposition.**


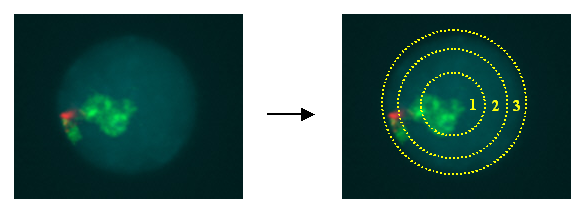


A. FISH analysis on interphase nuclei using WCPX (green) and WCPY (red). Each nucleus has been divided in three concentric circles of equal area (1-internal; 2-intermediate; 3-external, associated to the nuclear membrane) and then the position of derivative Y chromosome was evaluated. In this figure the derivative Y chromosome is located in the third shell in association to the nuclear membrane, a position which typical of the Barr body.


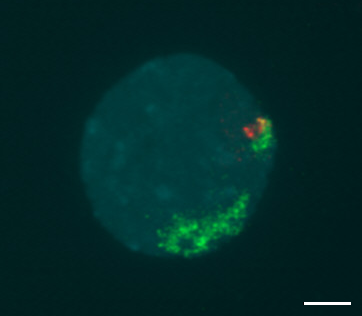


B. FISH on interphase nuclei using whole chromosome painting WCP X (green) and WCP Y (red) probes. The image shows the more condensed and regular shape of derivative Y chromosome in comparison to normal X chromosome, which is more irregular and spread. Derivative Y chromosome is located in association to the nuclear membrane. Bar=10μm.
