# Supplementary material for: Chromosome territories, X;Y translocation and Premature Ovarian Failure: is there a relationship?
Source: Mol Cytogenet. 2009 Sep 27;2:19. doi: 10.1186/1755-8166-2-19 (PMC2761935; doi:10.1186/1755-8166-2-19)
Supplement: Additional file 4 — Statistical analysis of chromosome territory (CT) and centromere distribution in interphase nuclei. A. The table summarise the statistical analysis (χ2 test) performed on the results of CT distances (X, Y and derivative Y chromosomes) in interphase nuclei. B. The table summarise the statistical analysis (χ2 test) performed on the results of X and Y centromere reciprocal distribution in interphase nuclei. [file 1755-8166-2-19-S4.DOC]

**Additional file 4**

**Statistical analysis of chromosome territory (CT) and centromere distribution in interphase nuclei.**

|  | **χ^2^ Test, p-value** | | | |
| --- | --- | --- | --- | --- |
|  | **CT Distance** | | | |
|  | **≤30%** | **30-50%** | **>50%** | **General** |
| **Male vs. Female Controls** | 0.935 | 0.662 | 0.770 | 0.876 |
| **Patient vs. Pooled Controls** | 0.924 | 0.104 | 0.155 | 0.184 |

A. Statistical analysis of chromosome territory (CT) distances (X, Y and derivative Y chromosomes) in interphase nuclei.

|  | **χ^2^ Test, p-value** | | | |
| --- | --- | --- | --- | --- |
|  | **CEP X; CEP Y Distance** | | | |
|  | **≤30%** | **30-50%** | **>50%** | **General** |
| **Patient vs. Male Controls** | 0.309 | 0.872 | 0.224 | 0.318 |

B. Statistical analysis of centromere distribution (X and Y centromeres) in interphase nuclei.
