# Supplementary material for: Chromosome territories, X;Y translocation and Premature Ovarian Failure: is there a relationship?
Source: Mol Cytogenet. 2009 Sep 27;2:19. doi: 10.1186/1755-8166-2-19 (PMC2761935; doi:10.1186/1755-8166-2-19)
Supplement: Additional file 5 — Chromosome territory image analysis. Graphic representation of chromosome territory image analysis. [file 1755-8166-2-19-S5.DOC]

**Additional file 5**

**
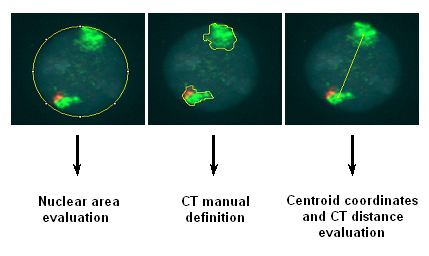
**

**Chromosome territory image analysis.** Analysis of the reciprocal position of chromosome territories (CT) in interphase nuclei. First nuclear area was evaluated comparing the nucleus to a circle of equal area; then sexual chromosome territories were manually defined; finally the program estimated the distance between the mass centroids of the two territories. The same method was used to evaluate centromeres reciprocal distribution.
