# Supplementary material for: Chromosome territories, X;Y translocation and Premature Ovarian Failure: is there a relationship?
Source: Mol Cytogenet. 2009 Sep 27;2:19. doi: 10.1186/1755-8166-2-19 (PMC2761935; doi:10.1186/1755-8166-2-19)
Supplement: Additional file 6 — Sequences of forward and reverse primers used in PCR reaction for SRY gene sequencing. Primer details for SRY sequencing. [file 1755-8166-2-19-S6.DOC]

**Additional file 6**

| **Primers** | **Sequence (5’-3’)** |
| --- | --- |
| **Forward** | GAATACATTGTCAGGGTACTAGG |
| **Reverse** | TCGTGTTGACACAACTTGTCTTG |
| **Forward_A_** | GAATACATTGTCAGGGTACTAGG |
| **Reverse_A_** | TTCTGTGCCTCCTGGAAGAATG |
| **Forward_B_** | GCGAAACTCAGAGATCAGCAAG |
| **Reverse_B_** | TCGTGTTGACACAACTTGTCTTG |

**Sequences of forward and reverse primers used in PCR reaction for SRY gene sequencing.** Primer details for SRY sequencing**.**
